# Surgical Strategies in Reoperation of the Proximal Aorta and Arch for Patients with Previous Frozen Elephant Trunk

**DOI:** 10.3390/jcm13144063

**Published:** 2024-07-11

**Authors:** Arian Arjomandi Rad, Ali Ansaripour, Dimitrios E. Magouliotis, Riccardo G. Abbasciano, Marinos Koulouroudias, Alessandro Viviano, Ulrich Rosendahl, Thanos Athanasiou, Antonios Kourliouros

**Affiliations:** 1 Medical Sciences Division, University of Oxford, Oxford OX3 9DU, UK; 2 Department of Cardiothoracic Surgery, John Radcliffe Hospital, Oxford University Hospitals NHS Foundation Trust, Oxford OX3 9DU, UK; ali.ansaripour@ouh.nhs.uk; 3 Department of Cardiothoracic Surgery, University Hospital of Larissa, School of Medical Sciences, 413 34 Larissa, Greece; dimitrios.magouliotis.18@ucl.ac.uk; 4 Department of Cardiothoracic Surgery, Hammersmith Hospital, Imperial College Healthcare NHS Trust, London W12 0HS, UKalessandro.viviano@nhs.net (A.V.); t.athanasiou@imperial.ac.uk (T.A.); 5 Trent Cardiac Centre, Nottingham University Hospitals, Nottingham NG5 1PB, UK; marinos.koulouroudias1@nhs.net; 6 Department of Cardiothoracic Surgery, Royal Brompton Hospital, Guy’s and St Thomas’ NHS Foundation Trust, London SW3 6NP, UK; u.rosendahl@rbht.nhs.uk

**Keywords:** frozen elephant trunk (FET), aortic surgery, reoperation

## Abstract

**Background:** The frozen elephant trunk (FET) technique is increasingly utilized for aortic arch replacement in cases of aortic dissections and aneurysms. This rise in usage has led to more patients needing redo aortic surgeries due to progression of existing conditions, FET-related complications, or new valvular/coronary diseases. This article aims to evaluate surgical techniques to minimize risks during these reoperations, including a case study of a complex redo surgery. **Methods:** A comprehensive examination of surgical strategies was conducted, focusing on preoperative preparation, cannulation site identification, cerebral and cardiac protective measures, and pitfalls to avoid. The importance of adapting to the modified anatomical landscape post-FET is emphasized. A detailed case study of a patient undergoing complex redo FET surgery is included. **Results:** The article identified key surgical strategies for reoperation in patients with prior FET, highlighting the importance of meticulous preoperative planning and execution. Techniques to minimize risks include detailed imaging for planning, strategic cannulation for optimal perfusion, multidisciplinary approaches as well as careful fail-safe measures. The case study demonstrates the practical application of these strategies in a high-risk scenario. The evidence underscores the necessity for individualized patient management and the development of standardized protocols. **Conclusions:** The FET technique, while effective for initial aortic arch repairs, often necessitates complex reoperations. Adopting advanced surgical strategies and multidisciplinary planning can significantly mitigate risks associated with these procedures. Future research should focus on refining these techniques and establishing standardized protocols to improve patient outcomes.

## 1. Introduction

**Figure 1:**
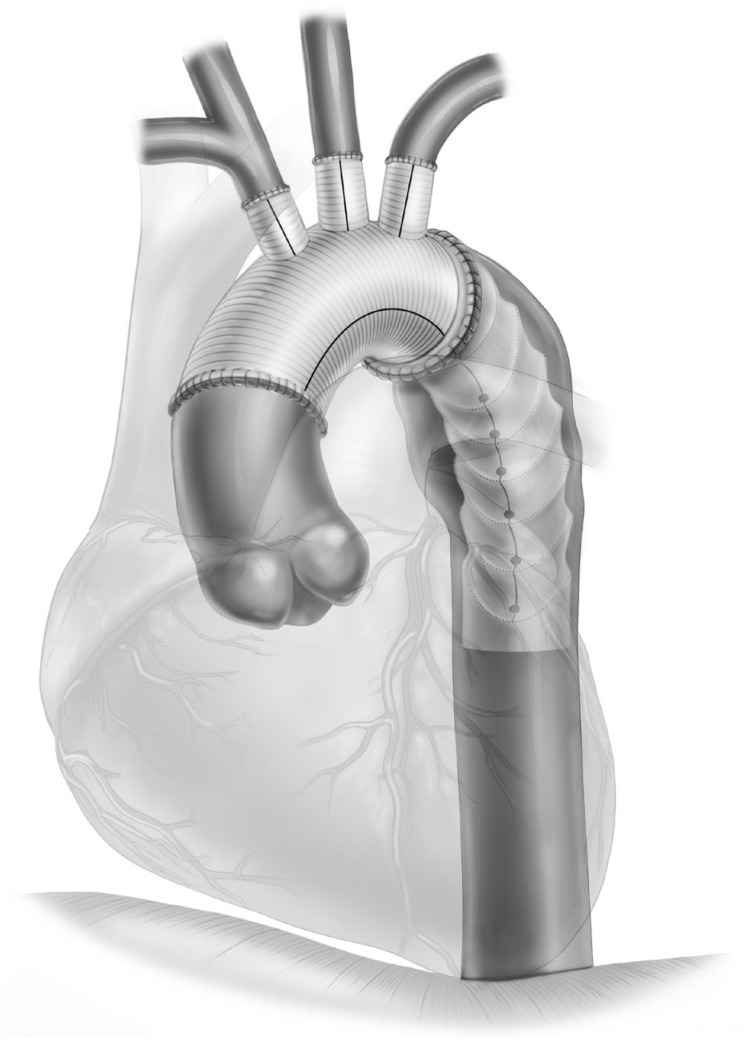
Illustration of the frozen elephant trunk (Thoraflex Hybrid) prosthesis in position.

The frozen elephant trunk (FET) graft technique has become the gold standard for aortic arch replacement in aortic dissections and aneurysms [1,2,3]. This technique has played a crucial role in the management of thoracic aortic diseases. Although the FET procedure (Figure 1) has demonstrated promise in promoting the favorable restructuring of aortic segments and presents the advantage of potentially being a one-step intervention, its long-term effectiveness, particularly in terms of the necessity for subsequent aortic reinterventions, continues to be a topic of ongoing investigation and discussion [4,5,6]. Additionally, its widespread uptake has led to several patients who develop de novo aortic disease, progression of residual pathology, or complications related to the presence of FET requiring reoperation.

Open surgical reoperations are occasionally required due to complications such as proximal anastomotic pseudoaneurysms, the enlargement of the aortic roots of marginal size that were initially preserved, new diagnoses of valve or coronary diseases that require surgical intervention, and extensive downstream aortic aneurysmatic dilatation or aortic dissection within the native thoracic aorta [7].

The process of reoperations is intricate and requires precise preparation and execution due to several aspects, such as changing anatomy, the possibility of re-entry harm, cannulation strategies in the context of previously utilized sites, and challenges associated with organ protection. This paper endeavors to explore the complexities associated with redo aortic surgeries in individuals who previously received FET implantation. This article describes the surgical strategies used to mitigate the risks associated with the reopening and reconstruction of the aorta in patients with FET.

## 2. Basics of the FET Procedure

The FET procedure represents a significant advancement in the surgical management of complex aortic diseases, particularly for conditions simultaneously affecting the aortic arch and the descending thoracic aorta [8]. Developed to address the limitations of the conventional elephant trunk technique and driven by innovations in endovascular technology, the FET procedure facilitates a comprehensive approach for treating extensive aortic pathologies. This technique integrates the replacement of parts or the entirety of the aortic arch with the antegrade delivery of a stent–graft into the descending aorta, thereby serving as both a definitive treatment for the arch and a preparatory step for any future endovascular interventions on the descending segment (Figure 1).

Indications for the FET procedure are broad yet specific, targeting acute aortic dissection, chronic aortic dissection, and chronic degenerative aneurysms of the aortic arch and descending thoracic aorta [9]. It is particularly valuable in acute Type A dissection, where it addresses mal-perfusion syndromes by stabilizing primary and re-entry tears, thereby promoting true lumen patency, and facilitating false lumen thrombosis [10]. The technique also offers an effective solution for chronic conditions, such as aneurysms involving the aortic arch and descending thoracic aorta, where it can prevent the progression of disease and reduce the risk of aortic rupture [2,11]. The FET procedure’s ability to provide a reliable proximal landing zone for subsequent thoracic endovascular aortic repair (TEVAR) significantly enhances the treatment strategy for these complex diseases, making it a pivotal tool in the aortic surgeon’s arsenal [12].

## 3. Further Planned Reintervention in Patients with FET

The evidence on aortic reinterventions following the FET procedure underscores the complex landscape of postoperative care and the nuanced approach required in managing thoracic aorta pathologies. The seminal study by Kreibich et al. (2020) revealed that a significant proportion (33%) of patients necessitated reinterventions after FET implantation, categorized into intended completion, anticipated reinterventions, and unexpected reinterventions [7]. That study highlighted the considerable risk of reintervention after FET, with rates escalating to 31% at 12 months, 49% at 24 months, and 64% at 36 months, emphasizing the procedure’s complexity and the critical need for vigilant postoperative follow-up [7].

Expanding on these findings, Di Marco et al. (2023) illustrated the frequent necessity and efficacy of TEVAR extensions following FET [13]. Their research, spanning over a decade, indicated that among 371 patients with FET, 119 required TEVAR extensions. Despite the seemingly high need for additional procedures, the outcomes were notably positive, with survival rates remaining high 1 to 10 years after extension. That study not only reflects the evolving landscape of aortic surgery but also reassures us regarding the safety and effectiveness of subsequent interventions after FET [13].

Furthermore, Folkmann et al. (2015) contributed valuable insights into the feasibility and success of second-stage thoracoabdominal (TA) aortic repairs following FET [14]. Their focused analysis on nine patients undergoing this subsequent intervention showed no in-hospital deaths or severe complications such as spinal cord ischemia or stroke, reinforcing the potential of FET not just as an immediate intervention but as a foundational procedure facilitating future necessary repairs [14].

Demal et al. (2021) reported the outcomes of 118 patient undergoing reoperation following FET, highlighting no mortality difference at 30 days between redo FET and primary FET (3.2% vs. 7.4%; *p* = 0.63) and 3-year mortality (22.2% vs. 16.7%; *p* = 0.72) [15]. Similar outcomes were also reported in terms of neurological dysfunction, paraplegia, and acute kidney failure.

Collectively, these studies paint a picture of FET as a pivotal yet initial step in the comprehensive management of complex aortic diseases. While FET offers a promising avenue for treating thoracic aorta pathologies, the evidence clearly illustrates the high likelihood of reinterventions, whether through endovascular, open, or hybrid approaches. These findings emphasize the necessity of individualized patient management strategies and the importance of a multidisciplinary approach for optimizing outcomes and navigating the intricacies inherent to post-FET care.

## 4. Indications for Redo FET

### 4.1. Endoleak with Sac Expansion

Redo FET procedures are critically indicated for managing persistent or recurrent endoleaks with sac expansion, highlighting the complexity of achieving a complete seal of the aortic repair. This condition, often a result of inadequate initial sealing or progression of the disease, significantly increases the risk of rupture. The presence of endoleaks, where blood flow persists outside the stent–graft lumen but within the aortic wall, poses significant risks, underscoring the need for precise surveillance and timely reoperation. The pathophysiological understanding of these conditions, including the potential for aneurysmal formations adjacent to or within previously treated segments due to ongoing degenerative processes or hemodynamic changes after repair, is crucial for planning effective redo FET interventions.

Kandola et al. (2020) reported a 28% incidence of endoleak and an 8% incidence of sac expansion among 36 single-stage procedures, highlighting the significance of these complications in FET procedures [16]. This was the most prevalent complication following FET reported by the authors. The research emphasized that patients without endoleak or sac expansion had stents that were >10% oversize and a >30 mm seal in the healthy aorta, suggesting that proper sizing and sealing are critical for minimizing complications [16]. In contrast, the frequency of Type 1A endoleak following novel FET techniques was reported to be about 8% in a study by Phùng et al., indicating the relatively low but significant risk of this complication [17].

### 4.2. Pseudoaneurysm/Anastomotic Leaks

The indication for redo FET in the context of pseudoaneurysms or anastomotic leaks after FET emphasizes the challenges posed by mechanical failures at surgical junctions. These complications necessitate complex dissection and reconstruction, reflecting the intricate nature of such interventions. Pseudoaneurysm formation following FET is a recognized postoperative complication, arising most commonly from stent–graft anastomosis failure [18]. Research by Martens et al. (2023) and Öz et al. (2021) has sheds light on the multifaceted nature of pseudoaneurysm formation following FET surgery [19,20]. Martens et al. (2023) explored the connection between intraluminal thrombus formation within the FET stent–graft and pseudoaneurysm development, suggesting that thrombus formation may signal or contribute to pseudoaneurysm risks, thereby highlighting the need for careful postoperative monitoring and potential technique refinement [19]. The lack of sufficient radial force and longitudinal stiffness in the hybrid graft, as discussed by Öz et al. (2021), can contribute to the complication, underlining the importance of selecting and sizing grafts appropriately to mitigate this risk [20]. Moreover, cases like that presented by Amirghofran et al. emphasize the potential for chronic erosion by sternal wires to lead to pseudoaneurysm formation, further complicating the postoperative landscape [21]. Denman et al. (2022) presented a case where mechanical trauma from a vent catheter during FET surgery led to left ventricular pseudoaneurysm formation, underlining the critical importance of meticulous intraoperative technique and instrument handling [22]. This evidence underscores the multifaceted nature of pseudoaneurysm formation after FET, necessitating diligent surgical planning, execution, and postoperative care to minimize patient risk and improve outcomes.

The incidence of anastomotic leak after FET at 10-year follow-up was reported by Ma et al. to be around 1% in a cohort of 518 patients receiving FET [23]. Interestingly, Pu et al. explored percutaneous aortic anastomosis leak closure after FET, reporting a success in achieving mild or lower leak grades in 90.6% of patients during midterm follow-up [24]. The decision regarding the surgical strategy, whether open, endovascular, or hybrid, is individualized based on pathology, feasibility, and patient characteristics, decided within a multidisciplinary setting to ensure optimal outcomes.

### 4.3. Infections including Fistulae Formation

Post-FET infections and fistulae formation, with their overt symptomatology, often require urgent intervention. These conditions represent critical indications for redo FET, demanding excision of the infected graft and careful reconstruction. The rate of infection after FET has not been widely reported, but data from small series indicate an incidence of around 2% in the perioperative period [25].

The absence of standardized protocols for the treatment of infections associated with hybrid FET prostheses is a critical issue highlighted by Nader et al. (2021), who advocated for a range of management strategies from surgical interventions such as debranching to the use of antibiotic therapy, aiming to circumvent the need for major redo surgeries [26].

A notable case reported by Varela Barca et al. involved a 47-year-old man who underwent emergency surgery due to an infected FET prosthesis caused by *Propionibacterium acnes* [27]. This case required the replacement of the graft and debranching of the three supra-aortic vessels, illustrating the surgical complexity and challenges posed by infections in such high-risk procedures. Similarly, Tsujimoto et al. (2021) described a unique case of graft infection with fungal vegetation on the FET, which was treated via anterolateral partial sternotomy, further emphasizing the rarity and intricacy of managing such infections [28]. Reineke et al. (2023) presented innovative single-stage, trans-sternal approaches for treating severe infections following FET surgery, exemplified through two cases [29]. Both cases involved patients who developed significant infections post-FET procedure, with one caused by *Streptococcus oralis* and the other by *Staphylococcus aureus*. The treatment involved the removal of the infected FET prosthesis and replacement with a custom-made valved conduit and a conventional elephant trunk constructed from bovine pericardium [29]. This method posed challenges, including considerable intimal damage to the descending aorta, which necessitated follow-up TEVAR procedures.

The pathophysiological foundation includes the consideration of graft-related complications, where infective complications can arise from material fatigue, infection, or mechanical failure. Addressing these challenges involves a multidisciplinary approach to guide perioperative management and surgical strategies, ensuring the removal of infected material while maintaining cardiovascular integrity.

### 4.4. Requirement for Additional Procedures in the Ascending Aorta/Root/Valves/Coronaries

The need for additional procedures in the context of redo FET due to disease progression or previous intervention failure requires a comprehensive understanding of the underlying disease, including aortic dissections and aneurysmal formations. Aortic dissections, particularly Type A treated initially with FET, may extend or experience expansion, necessitating further intervention. The management of such complexities involves considering the pathophysiological aspects of the disease, including distal stent–graft-induced new entry, failure of aortic remodeling, and the potential for graft degradation. The strategic approach involves reparative management techniques for resolving complications like kinking of the FET stent–graft; employing strategies such as total endovascular repair, open repair, and balloon dilatation; and deploying a second stent–graft, crucial for maintaining graft integrity and enhancing patient outcomes. Likewise, with the development of advanced percutaneous techniques for mitral and aortic valve disease, as well as complex percutaneous interventions for the coronaries, the need for redo surgery to address these pathologies in FET patients could decline. However, it should be noted that no current literature exists on the specific outcomes or reported cases of aortic valve replacement in this context, highlighting a significant gap in our understanding. This indicates a potential future avenue for research to better comprehend and manage these complex scenarios. For those occasions where open surgery is the only or the preferred option, a multidisciplinary approach, careful planning, and certain technical principles, which are presented in the following section, could assist with risk mitigation in this group of patients.

## 5. General Considerations for Redo Aortic Surgery

Redo aortic surgery presents a unique set of challenges that require advanced imaging techniques and innovative surgical approaches to ensure optimal patient outcomes. This section delves into these fundamental considerations, emphasizing the necessity of meticulous preoperative planning and the integration of multidisciplinary expertise. Given the complexity and high-risk nature of redo aortic surgery, particularly following previous interventions like FET procedures, understanding these critical aspects is paramount for successful management. This section discusses the prevalence and impact of adhesions, the necessity of alternative surgical approaches such as thoracotomy or hybrid methods, the importance of establishing peripheral arterial access, the challenges posed by prosthetic graft infections and pseudoaneurysm formations, and the vital role of advanced imaging techniques like CTA and MRI in preoperative planning and intraoperative decision making.

In redo aortic surgery, surgeons face a constellation of challenges that significantly impact the procedural approach and patient outcomes. Adhesions present a formidable barrier, with a reported prevalence as rate as high as 95% in patients undergoing surgery irrespective of anatomical location [30]. These fibrous bands not only increase the risk of intraoperative injury to the heart and great vessels but also complicate the dissection process, necessitating meticulous surgical planning and execution. The scenario of a “frozen chest” should also be considered, delineating a situation where traditional sternotomy may transition from being merely challenging to outright prohibitive. This necessitates the contemplation of alternative surgical avenues, such as thoracotomy or hybrid approaches, to mitigate the heightened risk of intraoperative complications. The mortality rates associated with redo aortic surgery vary depending on the number of prior surgeries a patient has undergone. Norton et al. found that patients undergoing redo aortic surgery after one previous operation had lower 30-day mortality rates than those undergoing surgery after multiple previous operations (12.3% vs. 21.7%, *p* = 0.03) [31].

**Figure 2:**
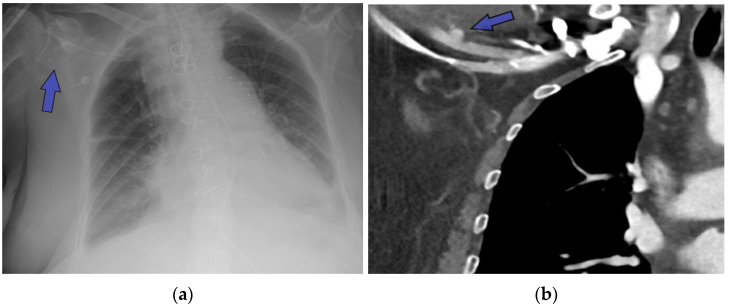

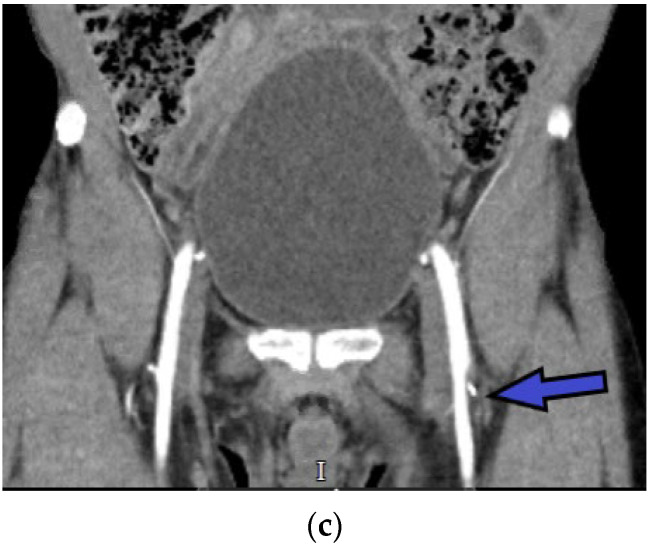
When establishment of peripheral arterial cannulation is considered, the use of imaging allows for identification hemostatic clips (**a**), vascular graft stumps (**b**), or iatrogenic injuries such as arterial stenosis (**c**) to identify the suitable remaining sites.

The proximity of the native ascending aorta or aortic graft to the sternum often necessitates the establishment of peripheral arterial access prior to redo sternotomy. Knowledge of previous access sites through the operation notes, clinical examination, and imaging is essential. While more technical details are presented in the section regarding specific surgical consideration in redo FET, presence of hemostatic clips (Figure 2a), vascular graft stumps (Figure 2b), or iatrogenic injuries such as arterial stenosis (Figure 2c) would inform the decision regarding access sites for safe peripheral cannulation.

The need for redo aortic surgery due to prosthetic graft infections occurs in approximately 2% of aortic surgeries [32]. These infections can lead to devastating outcomes, with a mortality rate of up to 8% to 66% depending on the type of graft; therefore, extensive surgical revision is needed, underscoring the importance of rigorous aseptic technique and, where applicable, the use of antibiotic-impregnated grafts during the original surgery [33].

**Figure 3:**
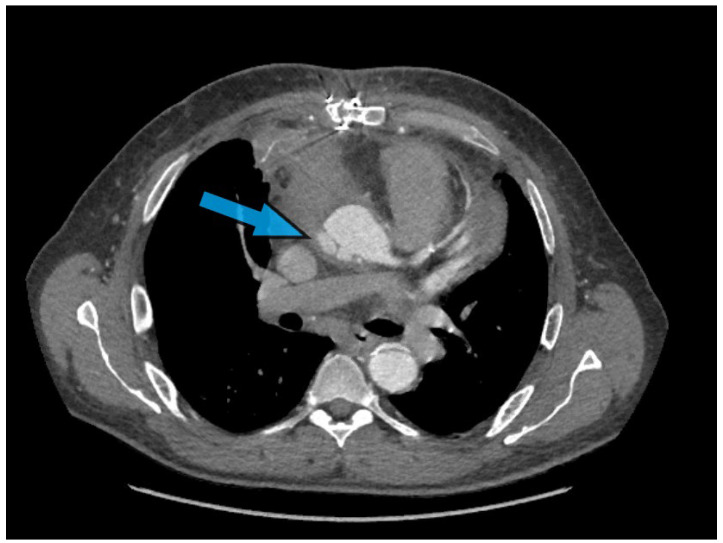
Computed tomography angiography (CTA) illustrating the presence of anastomotic pseudoaneurysm at the level of the sinotubular junction in a patient needing redo FET, highlighting the need to consider fail-safe strategies in order to contain major bleeding upon re-sternotomy.

Pseudoaneurysm formation after aortic surgery represents a perilous complication, arising in around 2–7% of cases [34,35]. These pseudoaneurysms pose a significant risk of rupture and hemorrhage (Figure 3), demanding prompt recognition and intervention. In-hospital mortality has been reported at 6.7% [36]. The challenge lies in the detection and differentiation of pseudoaneurysms from normal postoperative changes, which is where advanced imaging techniques play a pivotal role.

The utility of detailed imaging cannot be overstated in addressing these challenges. Preoperative computed tomography angiography (CTA) offers a comprehensive view of the aortic anatomy, adhesions, and the relationship of the graft to the surrounding structures, facilitating strategic surgical planning. Furthermore, CTA aids in the identification of pseudoaneurysms and their anatomical characteristics, guiding the surgical approach [37]. The downstream assessment of the aorta and peripheral vessels is essential for the selection of cannulation and perfusion strategies through CTA.

The utility of detailed imaging, particularly magnetic resonance imaging (MRI), complements the invaluable insights provided by CTA. MRI stands out for its superior soft tissue contrast and the ability to provide detailed images of the aortic wall and surrounding structures without the need for ionizing radiation. This modality is especially beneficial in evaluating the extent of prosthetic graft infections and in delineating the anatomy of pseudoaneurysms, their relationship to the native vessels, and the presence of surrounding fluid collections or abscesses [38,39]. Furthermore, MRI can assess aortic wall integrity and detect the subtle signs of infection or impending rupture that may not be evident on CTA [40]. It is important that these patients are discussed in a complex aortovascular MDT, and the surgical strategy must be agreed upon in advance. The requirement for vascular surgery input, imaging cardiology, interventional radiology, and anesthetic preassessment are also determined, and the patient must appropriately provide consent.

## 6. Specific Considerations for Redo Frozen Elephant Trunk Technique

### 6.1. Peripheral Cannulation Option

Peripheral cannulation strategies in redo FET surgery are crucial for ensuring optimal systemic and cerebral perfusion, particularly given the complexities and risks associated with reoperative aortic procedures. The selection of peripheral cannulation sites is guided by several key considerations, including the need to maintain uninterrupted cerebral perfusion, the accessibility of the vessels considering previous surgical interventions, and the specific requirements of the redo FET procedure. This discussion focuses on the primary peripheral cannulation sites—the axillary, femoral, and carotid arteries—highlighting their roles, advantages, and considerations in the context of redo FET surgery.

#### 6.1.1. Axillary Artery Cannulation

The axillary artery is the preferred site for arterial access in redo FET procedures, primarily due to its role in facilitating antegrade cerebral perfusion. This approach significantly reduces the risk of cerebral ischemia during periods of circulatory arrest or reduced flow states, which is a paramount concern in complex aortic arch surgeries [41,42]. The cannulation of the right axillary artery allows for the direct flow of oxygenated blood to the brain via the right subclavian and carotid arteries, maintaining cerebral perfusion even when the aortic arch is being operated on. Bilateral carotid perfusion is often recommended so direct left carotid cannulation can be performed from within the origin of the artery at the arch, when possible. A side vascular graft is often sewn to the axillary artery to facilitate cannulation, which preserves the integrity of the artery and ensures continuous blood flow to the upper limb [43]. The main advantages of axillary artery cannulation include a lower risk of embolic events, stable cerebral perfusion, and the possibility of initiating cardiopulmonary bypass before sternotomy. In addition, these vascular grafts can be interiorized into the chest in selected cases and serve as extra-anatomical bypasses when debranching of the head and neck vessels is essential.

#### 6.1.2. Femoral Artery Cannulation

The femoral artery serves as an alternative or adjunct site for establishing arterial inflow in redo FET surgery [41,42]. Its primary advantage lies in the ease and speed of access, making it a valuable option in emergency situations or when other cannulation sites are not viable. However, femoral artery cannulation is associated with a higher risk of retrograde embolization and may not provide optimal cerebral protection compared to antegrade strategies [44]. In the context of redo FET, femoral cannulation may be utilized to quickly establish cardiopulmonary bypass or as a part of a hybrid approach, where initial femoral access is used to stabilize the patient before transitioning to a more definitive cannulation strategy for selective brain perfusion.

#### 6.1.3. Carotid Artery Cannulation

Carotid artery cannulation, though less commonly employed, can be considered in specific redo FET scenarios where other peripheral sites are not accessible and when direct cerebral perfusion is required [45]. This technique involves the direct cannulation of the common carotid artery, offering a route for antegrade cerebral perfusion. Carotid cannulation is typically reserved for situations where axillary artery access is compromised or as part of a tailored approach to ensure cerebral protection in patients with complex aortic arch pathology or extensive vascular disease. The main considerations with carotid cannulation include the risk of local vascular injury and the potential for cerebral embolization, necessitating careful patient selection and technical expertise.

### 6.2. Preoperative Considerations When Assessing Peripheral Cannulation Options

**Figure 4:**
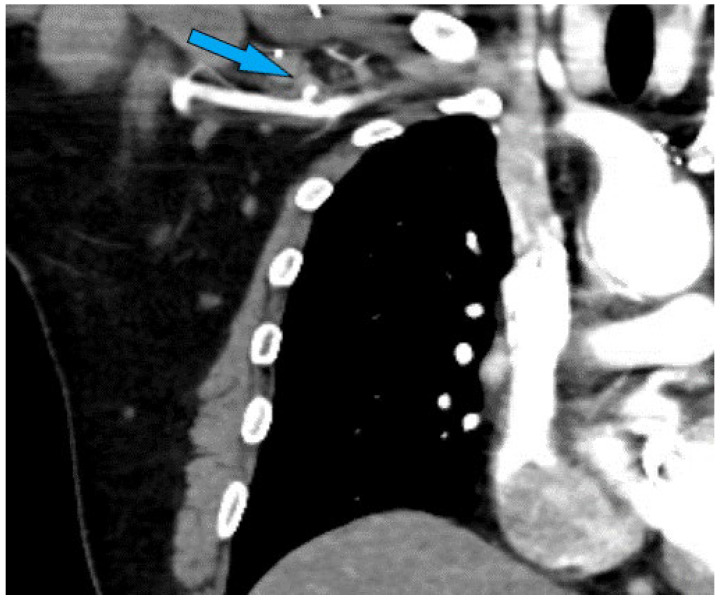
Axillary stump visualized on preoperative CTA indicating previous axillary artery cannulation with end-to-side graft.

High-resolution CT scans are invaluable in the preoperative assessment, offering detailed cross-sectional images of the thoracic cavity. These scans can identify the location and extent of aortic disease, the presence of calcifications, and the status of potential cannulation sites. CT angiography provides a detailed view of the aortic anatomy, enabling the surgical team to plan the cannulation strategy effectively. The visualization of a “stump” (Figure 4) in the axilla or other indicators of previous graft placements can significantly influence the choice of cannulation site, highlighting areas where caution is needed due to previous surgical alterations.

While not as detailed as CT scans, chest X-rays can still offer valuable insights into the overall thoracic anatomy, the position of the heart and aorta, and the presence of surgical clips from previous operations. These clips can serve as markers for previous surgical sites, guiding the surgeon in avoiding areas with dense adhesions or compromised vessel integrity.

**Figure 5:**
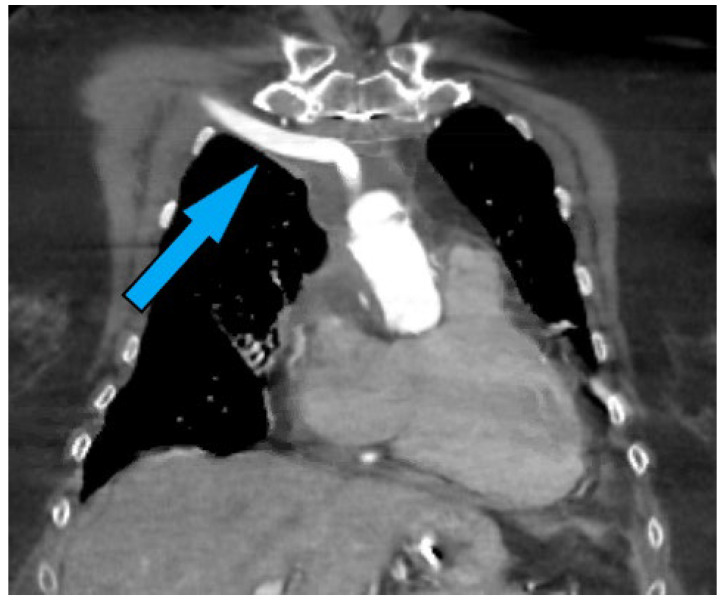
Illustration of extraanatomical bypass present in a patient with previous FET operation (right axillary artery to ascending aorta graft).

In the context of redo FET procedures, the challenge in accessing the chest in the presence of extensive adhesions or encountering an extra-anatomical bypass (Figure 5) from a previous intervention necessitates a nuanced approach. Surgeons may opt for peripheral cannulation as a precursor to sternotomy, thereby establishing CPB and ensuring hemodynamic stability before navigating the adhered mediastinum. This strategy not only facilitates safer entry into the chest but also mitigates the risk of inadvertent injury to vital structures [46].

### 6.3. Fail-Safe Measures for Unforeseen Complications

**Figure 6:**
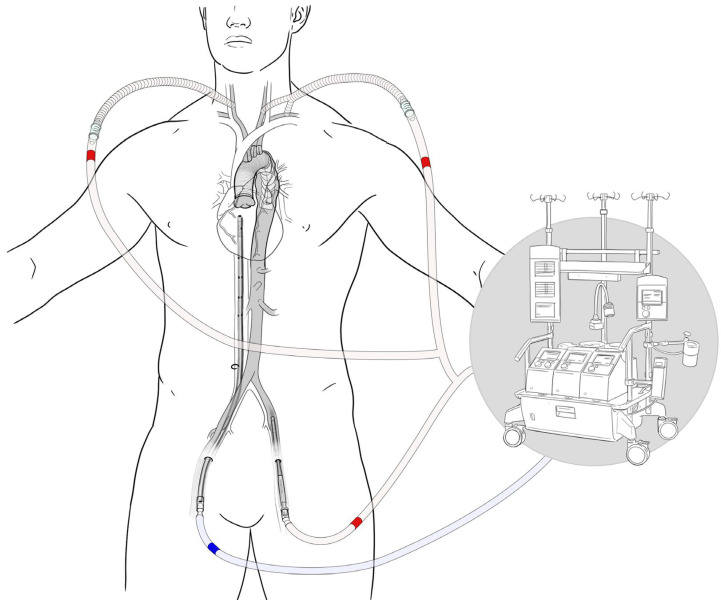
Illustration of fail-safe perfusion strategy involving venous drainage from the right femoral vein and arterial return via cannulas in both carotid arteries, and the common femoral artery.

Establishing a fail-safe perfusion strategy is paramount, particularly when navigating the potential complications associated with reoperative thoracic aortic procedures. The use of an absolute fail-safe approach, involving the cannulation of the right carotid artery, the left subclavian and left carotid arteries connected with a bifurcated graft, and the common femoral artery, all attached to the outflow of cardiopulmonary bypass (CPB), exemplifies a meticulous strategy designed to safeguard cerebral and lower body perfusion under adverse conditions (Figure 6). This configuration allows for the strategic placement of a snare around the descending aorta after limited thoracotomy, poised for immediate action. Should unexpected bleeding occur upon opening the aorta, the rapid clamping of the base of the carotid and subclavian arteries, in conjunction with the descending aorta, enables swift control of the hemorrhage, thereby mitigating the risk of catastrophic blood loss and ensuring the continuation of cerebral protection throughout this critical phase of the procedure.

Moreover, the integration of arterial femoral cannulation into this perfusion strategy underscores a comprehensive approach for maintaining systemic circulation, specifically to the lower body, during the surgery. The femoral artery’s role as an access point for CPB not only facilitates the management of overall blood flow but also serves as a crucial component of the fail-safe mechanism, ensuring that vital organs remain perfused even in the face of challenges encountered in the thoracic aorta. This dual-level approach, combining upper body cerebral perfusion management with lower-body systemic perfusion via the femoral artery, embodies a holistic strategy tailored to address the complexity and unpredictability inherent in redo FET surgeries.

Once the aorta is safely accessed and the necessary repairs or replacements are underway, the previously established bypass routes—specifically, those involving the right carotid, left subclavian, and any carotid–subclavian Y graft configurations—could be transitioned to serve as permanent conduits for blood flow. This approach effectively converts the temporary bypass setup, initially established for the purpose of ensuring cerebral and systemic perfusion during the critical phases of the operation, into a lasting solution that continues to provide targeted blood flow beyond the surgical intervention.

In cases where direct access to the chest is deemed too hazardous due to extensive adhesions or risk of injury to the heart and surrounding structures, the concept of veno-arterial extracorporeal membrane oxygenation (VA ECMO) as a bridge to safely establishing surgical access warrants consideration [47,48]. This modality not only supports cardiac and pulmonary function but also offers a controlled environment for navigating through adhesions or proceeding with complex dissections. Due to the lack of blood reservoir, the VA ECMO option is viable when the objective is cardiorespiratory support during difficult surgical dissections but not when the chances of significant bleeding are substantial.

### 6.4. Handling Existing Stent–Grafts

The presence of previously placed stent–grafts introduces several complexities to redo FET surgery [49,50]. One major concern is the risk of damaging the aortic intima during attempts to remove or manipulate these grafts. Given the delicate nature of the aortic wall, especially in a redo setting where previous interventions may have altered the structural integrity, the potential for intimal injury is a significant risk. This risk necessitates a careful assessment of the graft’s condition and position, as well as a judicious decision-making process regarding whether to remove, replace, or modify the existing graft. The techniques for managing these grafts include the careful dissection around the stent to preserve the integrity of the aortic wall and the use of endovascular procedures to reinforce or extend the existing graft without necessitating its removal [49,50]. Such approaches require a high level of expertise and familiarity with both open and endovascular techniques, underscoring the multidisciplinary nature of modern aortic surgery.

## 7. A Case of Complex Redo Frozen Elephant Trunk Surgery

### 7.1. Case Background

**Figure 7:**
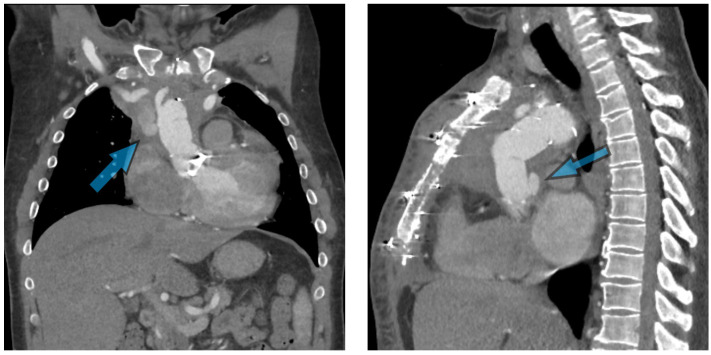
Preoperative CTA illustrating the presence of an extra-anatomical bypasses, chronic mediastinal infection, and pseudoaneurysm.

A patient in his 40s initially presented with chest pain, initially treated for pericarditis. A cardiac MRI revealed a Type A aortic dissection, leading to their first surgery for repair, including resuspension of the aortic valve and ascending aortic interposition graft with arch septectomy. Following this, they experienced complications, including a pseudoaneurysm at the proximal anastomosis of the ascending aorta, necessitating multiple redo surgeries. These included aortic valve replacement, ascending aorta replacement with a Gelweave (Terumo Aortic, Sunrise, FL, USA) graft, and installation of a Thoraflex Hybrid (Terumo Aortic, Sunrise, FL, USA) graft under deep hypothermic circulatory arrest three years later. Further complications led to additional interventions, including refashioning of an axillary extra-anatomical bypass and management of an infected sternal collection. Persistent issues, such as an infected pseudoaneurysm compressing the right axillary graft and a recurrent proximal aortic one, resulted in their referral for complex redo surgical intervention. The extensive medical history also included hypertension, hypercholesterolemia, atrial fibrillation, and recurrent postsurgery infections, complicating his treatment pathway (Figure 7).

### 7.2. Operative Approach

The surgical intervention began with bilateral neck dissection via supraclavicular incisions, aimed at isolating the carotid arteries. On the left, a vascular reconstruction was meticulously executed, involving the anastomosis of an 8 mm graft to a 10 mm Dacron graft in a Y-shaped configuration. This innovative grafting technique effectively connected the left subclavian artery with the left carotid artery, establishing a singular vascular outflow. On the right side, a thrombus was removed from the common carotid artery, subsequently ligating its origin and performing end-to-side anastomosis with a secondary outflow graft.

Attention was then shifted to establishing femoral access for cardiopulmonary bypass (CPB). The left groin was explored, isolating the femoral artery and vein. A 10 mm graft was anastomosed to the left femoral artery, ensuring a robust arterial line connection for CPB. Venous drainage was facilitated by the cannulation of the right femoral vein using a multistage cannula. A graphic example of this fail-safe cannulation strategy is shown in Figure 6. During the CPB, antegrade cerebral perfusion was meticulously maintained with cold blood perfusate administered through Dacron grafts. The perfusion process was closely monitored using near-infrared spectroscopy (NIRS) for cerebral flow and motor-evoked potentials (MEPs) for spinal cord integrity.

**Figure 8:**
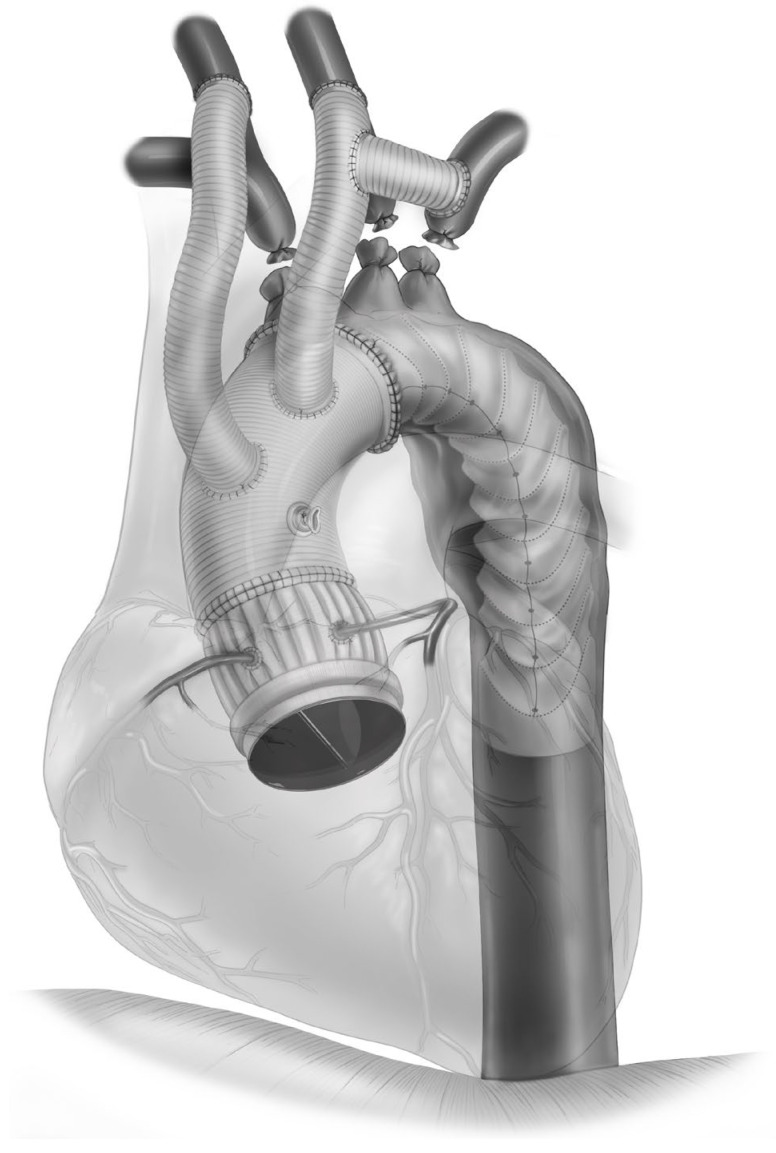
Illustration of the end result of a case of complex redo surgery with previous FET.

Concurrently, a left posterolateral thoracotomy provided access to the descending thoracic aorta. This strategic incision was part of a fail-safe strategy in case of unforeseen massive bleeding upon chest entry to be able to clamp or snare the descending aorta. A subsequent redo median sternotomy addressed the presence of dense adhesions and facilitated the management of an 8 × 10 cm pseudoaneurysm. Rigorous debridement was performed including ligation of previous extra-anatomical bypasses and removing compromised grafts. Cerebral perfusion was maintained throughout. The descending thoracic aorta was subsequently clamped, and a resection of neoascending aorta to the collar of the FET was performed, preserving the aortic root and mechanical valve. The stent–graft part of the FET did not appear infected. A 32 mm Gelweave (Terumo Aortic, FL, USA) graft was anastomosed distally to the FET and proximally to the sinotubular junction (STJ). The careful dissection and exclusion of the pseudoaneurysm’s neck during the proximal anastomosis were pivotal in preventing future complications. Tunneled Dacron vascular grafts from carotid arteries behind the neck were anastomosed to the ascending aorta to connect cerebral and body circulation. Routine closure was then performed. The final configuration at the end of this procedure is shown in Figure 8.

## 8. Current Evidence on Redo FET

The evidence surrounding the long-term outcomes and optimal management strategies following FET surgery remains scarce, primarily due to the operation’s technical complexity, uptake by specialist groups, and its relatively recent adoption in clinical practice. This scarcity underscores the need for ongoing research and robust data collection to better understand the procedure’s efficacy, potential complications, and patient selection criteria.

### 8.1. Reoperation Rates and Causes

Reoperation rates after FET surgery vary significantly across studies, reflecting the procedure’s complexity and the diverse patient populations. Ouzounian et al. (2020) observed a 7% in-hospital reoperation rate in their cohort, underscoring the critical nature of postoperative surveillance and the early identification of complications necessitating reoperation [51]. Dell’Aquila et al. (2017) demonstrated a significantly lower distal aortic reintervention rate of 22.5% in patients undergoing FET compared to 61.2% in those receiving conventional total arch replacement, highlighting the FET technique’s potential in reducing the need for subsequent aortic interventions [52]. A meta-analysis of 1279 patients who underwent the FET technique for managing acute Type A aortic dissection revealed a 9.6% chance of needing further surgery, with a notable 96.8% success rate in achieving false lumen thrombosis [53]. An updated review by Tian et al. (2020) showed that the rates of avoiding reintervention at 1, 3, and 5 years were 93.9%, 89.3%, and 86.8%, respectively, across a pool of 4178 patients from 37 studies investigating FET application under both elective and emergency scenarios [54].

### 8.2. Mortality Rates

The mortality associated with FET procedures is an essential metric for assessing its safety. A large systematic review by Tian et al. (2020) of 4178 patient undergoing FET identified that the overall 1-, 3-, and 5-year survival rates were 89.6%, 85.2%, and 82.0%, respectively [54]. In more complex scenarios, Luo et al. (2021) reported an operation mortality rate of 5.1% (4/79) in patients undergoing FET for chronic Type B or non-A non-B aortic dissection, indicating a relatively low immediate risk considering the procedure’s complexity. This rate is consistent with the outcomes observed in other complex aortic surgeries and underscores the FET technique’s efficacy in managing aortic dissections with an acceptable safety profile [55].

Despite the challenges, the FET procedure has demonstrated promising outcomes across various metrics. Tsumaru and Shimamoto (2023) reported no hospital deaths and minimal complications such as new strokes in their study on FET in ATAAD, highlighting the procedure’s potential for high success with low morbidity [56]. Similarly, the success rate in resecting or closing the proximal entry tear in Type A acute aortic dissections was higher with FET, with no cases of recurrent nerve palsy or paraplegia, as reported by Furutachi et al. (2019), suggesting an advantage in using FET for acute dissections [57]. Open redo surgery for FET is not common, and there are no large series that give robust mortality and morbidity outcomes. As such, these redo procedures should be carried out by specialist groups where a favorable volume/outcome relationship is more likely and risk stratification could be estimated on their existing experience and outcomes.

## 9. Conclusions

The FET technique represents a significant advance in the surgical management of thoracic aortic disease, embodying a sophisticated blend of open and endovascular approaches. As the evidence suggests, while FET has proven effective in addressing complex aortic pathologies, its deployment brings to the fore a series of critical surgical and operative considerations essential for optimizing patient outcomes. Key among these considerations is the meticulous planning of reoperations, which involves a nuanced understanding of the disease progression, the careful selection of cannulation sites to ensure optimal perfusion, and the strategic use of imaging modalities for preoperative planning and intraoperative guidance. These aspects are vital not only for the immediate success of the FET procedure but also for minimizing the likelihood and complexity of future interventions.

Moreover, the management of complications such as graft-related issues, pseudoaneurysm formation, and infection risks underscores the complexity of care following FET. Surgeons must navigate these challenges with a deep knowledge of the potential complications, employing a comprehensive approach that includes rigorous aseptic techniques, the judicious use of antibiotic-impregnated grafts, and the adoption of fail-safe measures for unforeseen complications. The strategic consideration of peripheral cannulation options, the careful handling of existing stent–grafts, and the provision for extensive aortic replacement through staged procedures illustrate the operative intricacies inherent in redo FET surgeries.

These considerations highlight the imperative for a multidisciplinary approach to patient management, incorporating the expertise of vascular surgeons, cardiologists, radiologists, and rehabilitation specialists. Collaboration across these specialties is crucial for tailoring the intervention to the patient’s specific pathology and physiological needs, thereby enhancing the safety and efficacy of the FET technique.

## Data Availability

All data are available from the corresponding author upon valid request.

## References

[1] Kreibich M., Berger T., Morlock J., Kondov S., Scheumann J., Kari F.A., Rylski B., Siepe M., Beyersdorf F., Czerny M. (2018). The frozen elephant trunk technique for the treatment of acute complicated Type B aortic dissection. Eur. J. Cardiothorac. Surg..

[2] Czerny M., Rylski B., Kari F.A., Kreibich M., Morlock J., Scheumann J., Kondov S., Südkamp M., Siepe M., Beyersdorf F. (2017). Technical details making aortic arch replacement a safe procedure using the Thoraflex^TM^ Hybrid prosthesis. Eur. J. Cardiothorac. Surg..

[3] Shrestha M., Bachet J., Bavaria J., Carrel T.P., De Paulis R., Di Bartolomeo R., Etz C.D., Grabenwöger M., Grimm M., Haverich A. (2015). Current status and recommendations for use of the frozen elephant trunk technique: A position paper by the Vascular Domain of, EACTS. Eur. J. Cardiothorac. Surg..

[4] Dohle D.S., Tsagakis K., Janosi R.A., Benedik J., Kühl H., Penkova L., Stebner F., Wendt D., Jakob H. (2016). Aortic remodelling in aortic dissection after frozen elephant trunk. Eur. J. Cardiothorac. Surg..

[5] Berger T., Kreibich M., Morlock J., Kondov S., Scheumann J., Kari F.A., Rylski B., Siepe M., Beyersdorf F., Czerny M. (2018). True-lumen and false-lumen diameter changes in the downstream aorta after frozen elephant trunk implantation. Eur. J. Cardiothorac. Surg..

[6] Luehr M., Peterss S., Zierer A., Pacini D., Etz C.D., Shrestha M.L., Tsagakis K., Rylski B., Esposito G., Kallenbach K. (2018). Aortic events and reoperations after elective arch surgery: Incidence, surgical strategies and outcomes. Eur. J. Cardiothorac. Surg..

[7] Kreibich M., Berger T., Rylski B., Chen Z., Beyersdorf F., Siepe M., Czerny M. (2020). Aortic reinterventions after the frozen elephant trunk procedure. J. Thorac. Cardiovasc. Surg..

[8] Tsagakis K., Osswald A., Weymann A., Demircioglu A., Schmack B., Wendt D., Jakob H., Ruhparwar A. (2022). The frozen elephant trunk technique: Impact of proximalization and the four-sites perfusion technique. Eur. J. Cardio-Thorac. Surg..

[9] Damberg A., Schälte G., Autschbach R., Hoffman A. (2013). Safety and pitfalls in frozen elephant trunk implantation. Ann. Cardiothorac. Surg..

[10] Chivasso P., Mastrogiovanni G., Miele M., Bruno V.D., Rosciano A., Montella A.P., Triggiani D., Colombino M., Cafarelli F., Leone R. (2021). Frozen Elephant Trunk Technique in Acute Type A Aortic Dissection: Is It for All?. Medicina.

[11] Papakonstantinou P.E., Benia D., Polyzos D., Papakonstantinou K., Rorris F.P., Toulgaridis F., Manousiadis K., Xydonas S., Sideris A. (2022). Chronic thoracic aortic dissection: How to treat, when to intervene. Life.

[12] Fortin W., Gautier C.H., Escande R., Bel A., Sutter W., El Batti S., Julia P., Achouh P., Alsac J.M. (2024). Thoracic Endovascular Repair after Total Aortic Arch Replacement with Frozen Elephant Trunk for Type a Aortic Dissection. Ann. Vasc. Surg..

[13] Di Marco L., Nocera C., Snaidero S., Campanini F., Buia F., Lovato L., Murana G., Pacini D. (2023). Staging TEVAR after FET—An exception or the rule?. Indian J. Thorac. Cardiovasc. Surg..

[14] Folkmann S., Weiss G., Pisarik H., Czerny M., Grabenwoger M. (2015). Thoracoabdominal aortic aneurysm repair after frozen elephant trunk procedure. Eur. J. Cardio-Thorac. Surg..

[15] Demal T.J., Bax L., Brickwedel J., Kölbel T., Vettorazzi E., Sitzmann F., Reichenspurner H., Detter C. (2021). Outcome of the frozen elephant trunk procedure as a redo operation. Interact. CardioVascular Thorac. Surg..

[16] Kandola S., Abdulsalam A., Field M., Fisher R.K. (2020). Frozen elephant trunk repair of aortic aneurysms: How to reduce the incidence of endoleak and reintervention. JTCVS Tech..

[17] Phung D.H., Nguyen T.S., Vo H.L., Vu N.T., Duong N.T., Pham V.L., Doan Q.H., Nguyen H.U. (2021). A novel modification of frozen elephant trunk technique: Unique protocol from one institution. Eur. Rev. Med. Pharmacol. Sci..

[18] Wada Y., Sakamoto K., Marui A., Ohno N. (2022). Pseudoaneurysm due to stentgraft–graft anastomosis failure: A case report. JTCVS Tech..

[19] Martens A., Beckmann E., Kaufeld T., Arar M., Natanov R., Fleissner F., Korte W., Krueger H., Boethig D., Haverich A. (2023). Features and risk factors of early intraluminal thrombus formation within the frozen elephant trunk stent graft. J. Thorac. Cardiovasc. Surg..

[20] Öz T., Prendes C.F., Stana J., Konstantinou N., Pichlmaier M., Tsilimparis N. (2021). A Case Report: Is the Lack of Sufficient Radial Force Unfreezing the “Frozen Elephant Trunk”?. J. Endovasc. Ther..

[21] Amirghofran A.A., Nirooei E., Ostovan M.A. (2021). Ascending aorta graft pseudoaneurysm and aortobronchial fistula caused by a fractured sternal wire: A case report. J. Cardiothorac. Surg..

[22] Denman E., Marvaki A., Huang M., Lamas S., Harrison J., Ammar T., Deshpande R., Monaghan M.J., Papachristidis A. (2022). Unexpected finding after aortic arch operation: A left ventricular pseudoaneurysm–Who is the culprit?. Echocardiography.

[23] Ma W.G., Chen Y., Zhang W., Li Q., Li J.R., Zheng J., Liu Y.M., Zhu J.M., Sun L.Z. (2020). Extended repair for acute type A aortic dissection: Long-term outcomes of the frozen elephant trunk technique beyond 10 years. J. Cardiovasc. Surg..

[24] Pu J., Song G., Ke Y., Ma X., Wu W., Zhang H. (2023). Techniques and outcomes of percutaneous aortic anastomosis leak closure after frozen elephant trunk procedure for aortic dissection. J. Thorac. Dis..

[25] Dahl T.S., Lindblom R.P. (2023). Intermediate outcomes following arch reconstruction with frozen elephant trunk, a single centre study. J. Cardiothorac. Surg..

[26] Nader J., Chabry Y., Nazih H., Caus T. (2021). Frozen elephant trunk infection: To defrost or to debranch?. Eur. J. Cardio-Thorac. Surg..

[27] Barca L.V., Esteban-Lucia L., Tomás-Mallebrera M., Pello-Lázaro A., Aldámiz-Echevarría G. (2023). Treatment For Infected Frozen Elephant Trunk Prosthesis Caused by *Propionibacterium acnes*: A Surgical Challenge. Braz. J. Cardiovasc. Surg..

[28] Tsujimoto T., Omura A., Inoue T., Chomei S., Hamaguchi M., Inoue T., Nakai H., Yamanaka K., Okada K. (2021). Anterolateral partial sternotomy for treatment of graft infection with fungal vegetation on the frozen elephant trunk: A case report. Ann. Vasc. Dis..

[29] Reineke D., Makaloski V., Schoenhoff F., Siepe M. (2023). Treatment of infected hybrid arch prosthesis with self-assembled bovine elephant trunk grafts. Eur. J. Cardio-Thorac. Surg..

[30] Fatehi Hassanabad A., Zarzycki A.N., Jeon K., Deniset J.F., Fedak P.W. (2021). Post-operative adhesions: A comprehensive review of mechanisms. Biomedicines.

[31] Norton E.L., Kalra K., Leshnower B.G., Wei J.W., Binongo J.N., Chen E.P. (2023). Redo aortic surgery: Does one versus multiple affect outcomes?. JTCVS Open.

[32] FitzGerald S.F., Kelly C., Humphreys H. (2005). Diagnosis and treatment of prosthetic aortic graft infections: Confusion and inconsistency in the absence of evidence or consensus. J. Antimicrob. Chemother..

[33] Kouijzer I.J., Baranelli C.T., Maat I., van den Heuvel F.M., Aarntzen E.H., Smith T., de Mast Q., Geuzebroek G.S. (2023). Thoracic aortic vascular graft infection: Outcome after conservative treatment without graft removal. Eur. J. Cardio-Thorac. Surg..

[34] Katsumata T., Moorjani N., Vaccari G., Westaby S. (2000). Mediastinal false aneurysm after thoracic aortic surgery. Ann. Thorac. Surg..

[35] Katzenschlager R., Ugurluoglu A., Ahmadi A., Hülsmann M., Koppensteiner R., Larch E., Maca T., Minar E., Stümpflen A., Ehringer H. (1995). Incidence of pseudoaneurysm after diagnostic and therapeutic angiography. Radiology.

[36] Atik F.A., Navia J.L., Svensson L.G., Vega P.R., Feng J., Brizzio M.E., Gillinov A.M., Pettersson B.G., Blackstone E.H., Lytle B.W. (2006). Surgical treatment of pseudoaneurysm of the thoracic aorta. J. Thorac. Cardiovasc. Surg..

[37] Latson Jr L.A., DeAnda Jr A., Ko J.P. (2017). Imaging of the postsurgical thoracic aorta: A state-of-the-art review. J. Thorac. Imaging.

[38] Koulouroudias M., Velissarios K., Kokotsakis J., Magouliotis D.E., Tsipas P., Arjomandi Rad A., Viviano A., Kourliouros A., Athanasiou T. (2023). Sizing the Frozen Elephant Trunk Based on Aortic Pathology and the Importance of Pre-Operative Imaging. J. Clin. Med..

[39] Son A.Y., Jarvis K., Markl M., Malaisrie S.C. (2021). 4D flow MRI after aortic replacement with frozen elephant trunk using thoraflex hybrid graft. J. Card. Surg..

[40] Rajiah P. (2013). CT and MRI in the evaluation of thoracic aortic diseases. Int. J. Vasc. Med..

[41] Kusadokoro S., Kimura N., Hori D., Hattori M., Matsunaga W., Itagaki R., Yuri K., Mieno M., Nakamura M., Yamaguchi A. (2020). Utility of double arterial cannulation for surgical repair of acute type A dissection. Eur. J. Cardiothorac. Surg..

[42] Liang S., Liu Y., Zhang B., Dun Y., Guo H., Qian X., Sun X. (2022). Cannulation strategy in frozen elephant trunk for type A aortic dissection: Double arterial cannulation approach. Eur. J. Cardiothorac. Surg..

[43] Sabik J.F., Nemeh H., Lytle B.W., Blackstone E.H., Gillinov A.M., Rajeswaran J., Cosgrove D.M. (2004). Cannulation of the axillary artery with a side graft reduces morbidity. Ann. Thorac. Surg..

[44] Choudhary S.K., Reddy P.R. (2022). Cannulation strategies in aortic surgery: Techniques and decision making. Indian. J. Thorac. Cardiovasc. Surg..

[45] Suzuki R., Akita M., Miyazaki S., Shimano R. (2023). Extra-anatomical left common carotid and subclavian artery bypass followed by aortic arch replacement with frozen elephant trunk. J. Cardiothorac. Surg..

[46] Luciani N., Anselmi A., De Geest R., Martinelli L., Perisano M., Possati G. (2008). Extracorporeal circulation by peripheral cannulation before redo sternotomy: Indications and results. J. Thorac. Cardiovasc. Surg..

[47] Hou J.Y., Wang C.S., Lai H., Sun Y.X., Li X., Zheng J.L., Wang H., Luo J.C., Tu G.W., Luo Z. (2021). Veno-Arterial Extracorporeal Membrane Oxygenation for Patients Undergoing Acute Type A Aortic Dissection Surgery: A Six-Year Experience. Front. Cardiovasc. Med..

[48] Fan F., Zhou Q., Pan J., Cao H., Li K., Xue Y., Ge M., Luo X., Chen Y., Wang D. (2021). Clinical outcomes of postoperative extracorporeal membrane oxygenation support in Stanford type a aortic dissection. BMC Anesth..

[49] Sato S., Nakamura A., Shimizu Y., Goto T., Kitahara A., Koike T., Okamoto T., Tsuchida M. (2019). Early and mid-term outcomes of simultaneous thoracic endovascular stent grafting and combined resection of thoracic malignancies and the aortic wall. Gen. Thorac. Cardiovasc. Surg..

[50] Leeuwerke S.J.G., Bohan P., Saleem B.R., Clucas J., Reijnen M.M.P.J., Zeebregts C.J. (2022). Frozen Elephant Trunk Completion: Endovascular Extension in The Distal Thoracic Aorta. Surg. Technol. Int..

[51] Ouzounian M., Hage A., Chung J., Stevens L.M., El-Hamamsy I., Chauvette V., Dagenais F., Cartier A., Peterson M., Harrington A. (2020). Hybrid arch frozen elephant trunk repair: Evidence from the Canadian Thoracic Aortic Collaborative. Ann. Cardiothorac. Surg..

[52] Dell’Aquila A.M., Pollari F., Fattouch K., Santarpino G., Hillebrand J., Schneider S., Landwerht J., Nasso G., Gregorini R., Del Giglio M. (2017). Early outcomes in re-do operation after acute type A aortic dissection: Results from the multicenter REAAD database. Heart Vessel..

[53] Takagi H., Umemoto T., ALICE Group (2016). A meta-analysis of total arch replacement with frozen elephant trunk in acute type A aortic dissection. Vasc. Endovasc. Surg..

[54] Tian D.H., Ha H., Joshi Y., Yan T.D. (2020). Long-term outcomes of the frozen elephant trunk procedure: A systematic review. Ann. Cardiothorac. Surg..

[55] Luo C., Qi R., Zhong Y., Chen S., Liu H., Guo R., Ge Y., Sun L., Zhu J. (2021). Early and long-term follow-up for chronic type B and type non-A non-B aortic dissection using the frozen elephant trunk technique. Front. Cardiovasc. Med..

[56] Tsumaru S., Shimamoto T. (2023). Abstract 15941: Efficacy of Frozen Elephant Trunk for Acute Type A Aortic Dissection Circulation. Circulation.

[57] Furutachi A., Takamatsu M., Nogami E., Hamada K., Yunoki J., Itoh M., Kamohara K. (2019). Early and mid-term outcomes of total arch replacement with the frozen elephant trunk technique for type A acute aortic dissection. Interact. Cardiovasc. Thorac. Surg..

